# Imaging the Ion–Molecule Reaction Dynamics of O^–^ + CD_4_

**DOI:** 10.1021/acs.jpca.3c08274

**Published:** 2024-04-10

**Authors:** Atilay Ayasli, Petra Tóth, Tim Michaelsen, Thomas Gstir, Fabio Zappa, Dóra Papp, Gábor Czakó, Roland Wester

**Affiliations:** † Institut für Ionenphysik und Angewandte Physik, Universität Innsbruck, Technikerstraße 25, Innsbruck 6020, Austria; ‡ MTA-SZTE Lendület Computational Reaction Dynamics Research Group, Interdisciplinary Excellence Centre and Department of Physical Chemistry and Materials Science, Institute of Chemistry, University of Szeged, Rerrich Béla tér 1, Szeged H-6720, Hungary

## Abstract

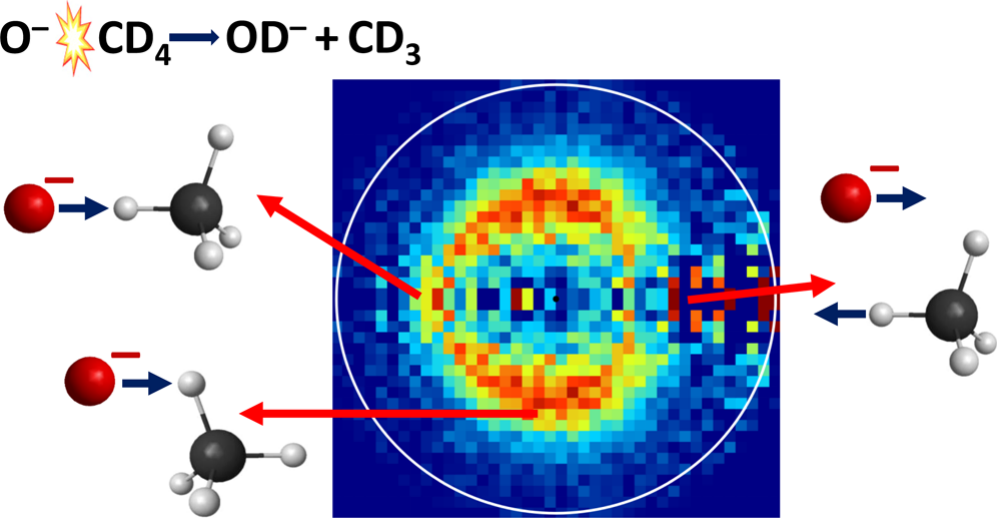

While neutral reactions involved in methane oxidation have been intensively studied, much less information is known about the reaction dynamics of the oxygen radical anion with methane. Here, we study the scattering dynamics of this anion–molecule reaction using crossed-beam velocity map imaging with deuterated methane. Differential scattering cross sections for the deuterium abstraction channel have been determined at relative collision energies between 0.2 and 1.5 eV and ab initio calculations of the important stationary points along the reaction pathway have been performed. At lower collision energies, direct backscattering and indirect complex-mediated reaction dynamics are observed, whereas at higher energies, sideways deuterium stripping dominates the reaction. Above 0.7 eV collision energy, a suppressed cross section is observed at low product ion velocities, which is likely caused by the endoergic pathway of combined deuteron/deuterium transfer, forming heavy water. The measured product internal energy is attributed mainly to the low-lying deformation and out-of-plane bending vibrations of the methyl radical product. The results are compared with a previous crossed-beam result for the reaction of oxygen anions with nondeuterated ¸methane and with the related neutral–neutral reactions, showing similar dynamics and qualitative agreement.

## Introduction

The study of bimolecular reaction dynamics of gas-phase reactions gives insights in how ions, atoms, and molecules interact during processes of chemical change.^1^ It provides a means to probe how they rearrange during a chemical process and how available energy is partitioned between products. Reactions of atoms with diatomic molecules have given us a deep understanding on elementary chemical processes on the molecular scale, starting with Polanyi’s rules of reaction dynamics^2^ and culminating in full quantum state-to-state-resolved dynamics.^3,4^ Moving beyond collisions of three atoms into polyatomic reactions, the dynamics of a reaction become increasingly complicated through additional degrees of freedom of vibrational and rotational motion. An investigation of reactions with methane CH_4_/CD_4_ offers an important model system to investigate the polyatomic reaction dynamics. The dynamics of methane with neutral atoms have been extensively researched over the years both experimentally^5−13^ and theoretically.^14−17^ This has provided a detailed understanding of its reactivity on the molecular level and has made it possible to determine the role of excited vibrational motion, particularly if vibrational excitation actively promotes a reaction or acts as a spectator.

Contrary to the extensive work on neutral collisions, the dynamics of ion–molecule reactions with methane have remained much less studied. In this article, we focus on reactive scattering of the radical anion O^–^ with deuterated methane CD_4_. O^–^, acting as a strong base, can abstract a deuterium in the slightly exothermic reaction
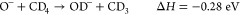
1

This abstraction mechanism is key in the ionic paths of lean oxygen methane combustion^18,19^ and atmospheric chemistry.^20^ The reaction rate coefficient for O^–^ with CH_4_ has been measured a long time ago by Lindinger et al.^21^ This result showed an increase in reactivity with increasing collision energy. From a thermal rate constant of 7 × 10^–11^ cm^3^ s^–1^, the rates increased to 2 × 10^–10^ cm^3^ s^–1^ at 1 eV. An exploration of the temperature-dependent rates for reaction 1, including other isotopologues of methane, was conducted by Viggiano et al.^22^ They reported a similar increase in reactivity with increasing temperature, but also found a strong kinetic isotopic effect in comparison to CH_4_. As for the dynamics of this system, Carpenter and Farrar^23^ measured product energy and angular distributions of the hydrogen abstraction in O^–^ + CH_4_. Their findings concluded that this reaction proceeds predominantly via a direct mechanism. This is supported by the recent on-the-fly molecular dynamics simulations, which found predominantly direct dynamics at collision energies above 1.5 eV.^24^

Here, we present experimental reactive scattering data for reaction 1 using a velocity map imaging (VMI) spectrometer in combination with the crossed-beam technique. We obtained experimental energy- and angle-dependent differential scattering cross sections for the OD^–^ product anion in the energy range of 0.2–1.5 eV relative collision energy. Compared with previous experimental work by Carpenter and Farrar, the use of a VMI spectrometer allows us to also capture slow product ions formed in reactive collisions, which helps us to develop a complete dynamical picture of the abstraction mechanism.

## Methods

### Experimental Methods

The experimental data was recorded using a crossed-beam setup in combination with a VMI spectrometer.^25^ This technique allows us to record angle- and velocity-dependent differential cross sections of ionic products resulting from reactive ion–molecule scattering.^26,27^ Both experimental setup and the data analysis procedure have been described previously and only a brief description will be outlined here.^28,29^

A precursor gas mixture of 1–2% N_2_O seeded in argon was used during the experiment. O^–^ ions were created via dissociative electron attachment in a pulsed plasma-discharge source using a home-built piezoelectric cantilever valve. The ions were subsequently extracted perpendicularly with regard to the initial direction and were mass selected using a Wiley–McLaren-type electrode configuration for time-of-flight discrimination. Ion packets were guided into an octupole radiofrequency ion trap using a combination of einzel lenses and deflectors, where they were trapped for 40 ms during which time they were thermalized with room-temperature helium buffer gas. As the radical character of O^–^ makes it a highly reactive species, trace amounts of water in the gas lines and ion source chamber can lead to significant levels of OH^–^ in the pulsed ion beam. In addition, isotopic contaminations of ^17^O^–^ and ^18^O^–^ were part of the ion beam. To suppress these unwanted ion masses, we implemented a switched Bradbury–Nielsen mass gate^30^ between the ion source and the ion trap.

Ions were then extracted from the trap, decelerated to desired kinetic energies, and crossed under a laboratory angle of 60° with a skimmed expansion of pulsed neat CD_4_ (99% purity, backing pressure about 1.5 bar) inside the VMI spectrometer. The differentially pumped neutral beam chamber has recently been redesigned and now allows for higher backing pressures and translational alignment of the valve-to-skimmer distance during operation of the experiment.

Product anions were detected by using a microchannel plate detector coupled with a phosphor screen, where the position of an impact on the detector was recorded using a CCD camera. We additionally used a photomultiplier tube to record the flight time of the product ion, from which we can extract the velocity component along the axis perpendicular to the collision plain of the experiment. This gave us a kinematically complete (*v*_*x*_^lab^, *v*_*y*_^lab^, *v*_*z*_^lab^) measurement of each product ion in the laboratory frame. Using the reactant beam velocities, which were measured separately, the product velocity vectors were transformed in the center-of-mass frame. From these new vectors (*v*_*x*_, *v*_*y*_, *v*_*z*_), with *x*-axis parallel to the relative velocity axis, different variables of interest were calculated, such as the scattering angle θ or the velocity  perpendicular to the relative velocity axis. When filling the two-dimensional histograms of *v*_r_ vs *v*_*x*_, each event was weighted with 1/*v*_r_ to create an image that represented a slice through the three-dimensional product velocity distribution. The experiment was operated at a 20 Hz repetition rate in the single collision regime.

Experimental energy- and angle-dependent differential cross sections of OD^–^ product ions were recorded at five different relative collision energies ranging from 0.2 to 1.5 eV. Average pulsed beam widths (fwhm) of O^–^ ions and CD_4_ were 204 and 88 meV, respectively, resulting in a mean relative collision energy uncertainty of about 140 meV.

### Computational Methods

We characterized the stationary points of the H/D-abstraction pathways and various product channels of the O^–^(^2^P) + CH_4_/CD_4_ reactions by using high-level ab initio methods. Initial geometry optimizations and harmonic vibrational frequency computations were performed using the restricted (R) or unrestricted (U) second-order Møller–Plesset (MP2) method^31^ (see more details in the Supporting Information), with the correlation-consistent aug-cc-pVDZ basis set.^32^ To improve the description of electron correlation and basis-set convergence, we also used the restricted open-shell Hartree–Fock-based (ROHF) explicitly-correlated unrestricted coupled-cluster singles, doubles, and perturbative triples method (UCCSD(T)-F12b)^33^ with the aug-cc-pVDZ and aug-cc-pVTZ basis sets. At the UCCSD(T)-F12b/aug-cc-pVnZ [n = D and T] levels, we computed optimized structures and energies with the corresponding vibrational frequencies to obtain the classical and zero-point-energy-corrected vibrationally adiabatic relative energies for the O^–^ + CH_4_ reaction. At the highest UCCSD(T)-F12b/aug-cc-pVTZ level (unless otherwise noted; see the Supporting Information), we also determined the vibrational frequencies using the mass of the deuterium to obtain the adiabatic relative energies for the O^–^ + CD_4_ system. In order to ensure that ROHF converges to the lowest-energy solution, we applied the ManyHF method,^34^ which uses several different initial guess orbitals to find lower-energy Hartree–Fock references. In the most problematic case (TS), we performed ManyHF-based UCCSD(T)-F12b/aug-cc-pVTZ as well as Davidson-corrected^35^ multireference configuration interaction^36^ (MRCI+Q) single-point energy computations with the aug-cc-pVTZ basis set. For MRCI, an active space of 5 electrons in 3 spatial orbitals was used. All of the correlation methods utilized the frozen-core approach; i.e., only the valence electrons were correlated. We used the ab initio program packages MOLPRO 2015.1^37^ and 2023.2^38^ for the computations. Spin–orbit effects were not considered in the present study, because they were negligible at the present experimental resolution as the splitting between the excited ^2^P_1/2_ and ground ^2^P_3/2_ states of O^–^ is only 0.02 eV.

## Results and Discussion

**Figure 1:**
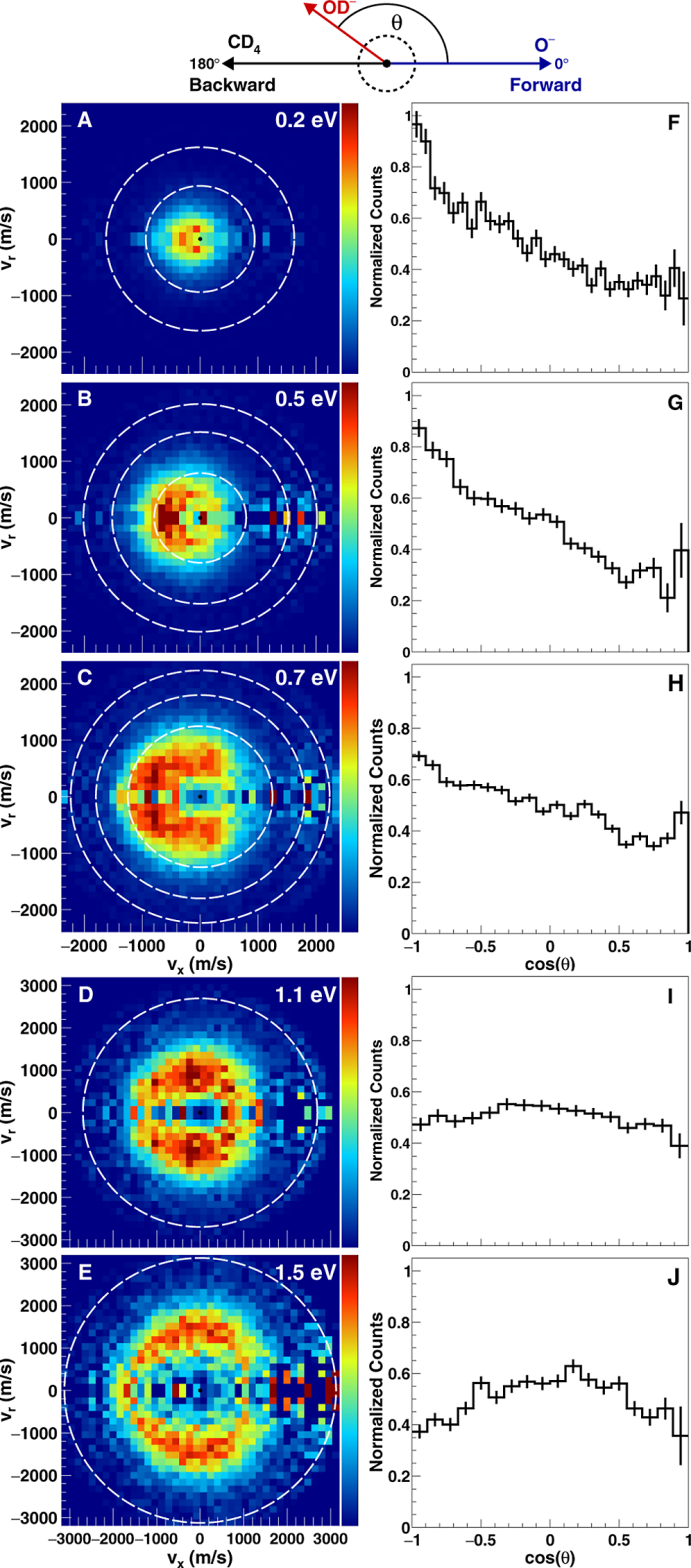
Experimental differential scattering cross sections (panels A–E) and extracted angular distributions (panels F–J) for the deuterium transfer reaction of O^–^ + CD_4_ at collision energies from 0.2 to 1.5 eV in the center-of-mass frame. The Newton diagram on the top depicts the relative orientation of both reactant velocity vectors. The outermost circles mark the kinematic cutoff for internal ground-state reaction products, while the inner dashed circles indicate the energies corresponding to the lowest excited vibrational levels of OD^–^(*v* = 1, 2). Note the different velocity scale in the lower two images.

The main results of the experiment are presented in Figure 1. Columns A–E show differential cross sections of the product OD^–^ in the center-of-mass frame of reference observed at various relative collision energies (see the Experimental Methods section). The Newton diagram on the top depicts the relative orientation of both reactants. In the chosen frame of reference, an incoming O^–^ is moving from left to right, while the neutral CD_4_ is moving from right to left. The direction of O^–^ defines our forward direction, which corresponds to the right-hand side of the images. Consequently, the left-hand side of the images shows backscattered products. The horizontal axis *v*_*x*_ is oriented along the collision axis (relative velocity vector) in the center-of-mass frame, while the vertical axis *v*_r_ shows the radial velocity component, which follows the cylindrical symmetry around the collision axis. The outermost dashed ring in all images represents the kinematic cutoff for the reaction, i.e., the maximum velocity a product can have. It is calculated from the total available energy, *E*_coll_ – Δ*H*, with *E*_coll_ and Δ*H* being the relative collision energy and reaction enthalpy, respectively. The inner rings in the differential cross section images mark excited vibrational levels of OD^–^ (first- and second-order vibrational constants ω_e_ = 337.7 meV and ω_e_*x*_e_ = 6.2 meV),^39^ with the outermost ring representing ν = 0. Panels F–J are angular distributions extracted from the cross sections and are matched in forward–backward directionality to the respective images.

Starting at the lowest collision energies of 0.2 and 0.5 eV (Figure 1A,B), we are able to detect two different reaction mechanisms. There is a pronounced direct backward scattered signature at both collision energies, where the OD^–^ product travels in the opposite direction of incoming O^–^. Angular deflection near 180° implies small impact-parameter events that can be assigned to a rebound-like mechanism.^40^ Specifically, we attribute this mechanism to a collinear [O–D–CD_3_]^−^ transition-state (TS) approach of O^–^ with one of the deuterium atoms in CD_4_, which then results in a direct rebound of the incident O^–^. The second mechanism is an indirect scattering signature where OD^–^ products are isotropically scattered. This is indicative for the formation of a complex involving both reactants, with an average lifetime longer than its rotational period. The dissociation of such a complex leads to isotropic scattering. The indirect mechanism is observed at 0.2 eV and to a lesser degree at 0.5 eV collision energy, but not at higher energies. Both backward and indirect mechanisms can be seen more quantitatively in the angular distributions in panels F and G of Figure 1, with reduced indirect scattering at 0.5 eV compared to 0.2 eV.

The intermediate collision energy of 0.7 eV (panel C) shows an onset of the missing flux, forming a ring at low product velocities. This indicates that indirect scattering through complex formation is suppressed. The observed rebound mechanism (backward scattering), stemming from a collinear approach of reactants at less than 0.5 eV energy, is also reduced in intensity. This can be seen more quantitatively in panel H. We instead observe sideways stripping and a direct forward-scattering stripping mechanism. Sideways stripping is the more prominent feature in the differential cross section, which we attribute to collisions of O^–^ with one of the three off-axis deuteriums of CD_4_ resulting in high-angle (approximately 90°) deflection of products. These events can be classified as intermediates to large impact-parameter events.^41^ Less pronounced is direct stripping toward the forward direction (right hemisphere). Stripping mechanisms can be attributed to large impact-parameter events, where an incoming O^–^ removes a deuterium at larger distances following little angular deflection of OD^–^ with respect to the incident O^–^.

At the two highest collision energies of 1.1 and 1.5 eV, indirect mechanisms vanish completely as the ring opens up due to the missing flux. All direct mechanisms mentioned above, i.e., small impact-parameter rebound, intermediate impact-parameter sideways scattering, and large impact-parameter direct forward-scattering stripping, are present at these energies. A direct comparison of the angular distributions (panels F–J) in Figure 1 shows that backscattered products are reduced in intensity with increasing collision energy (panels F–H), while the predominantly observed mechanism at energies above 1.0 eV is sideways scattering (panels I–J). This is in very good agreement with the on-the-fly molecular dynamics simulations, which predict direct dynamics and predominantly sideways scattering at this energy.^24^

**Figure 2:**
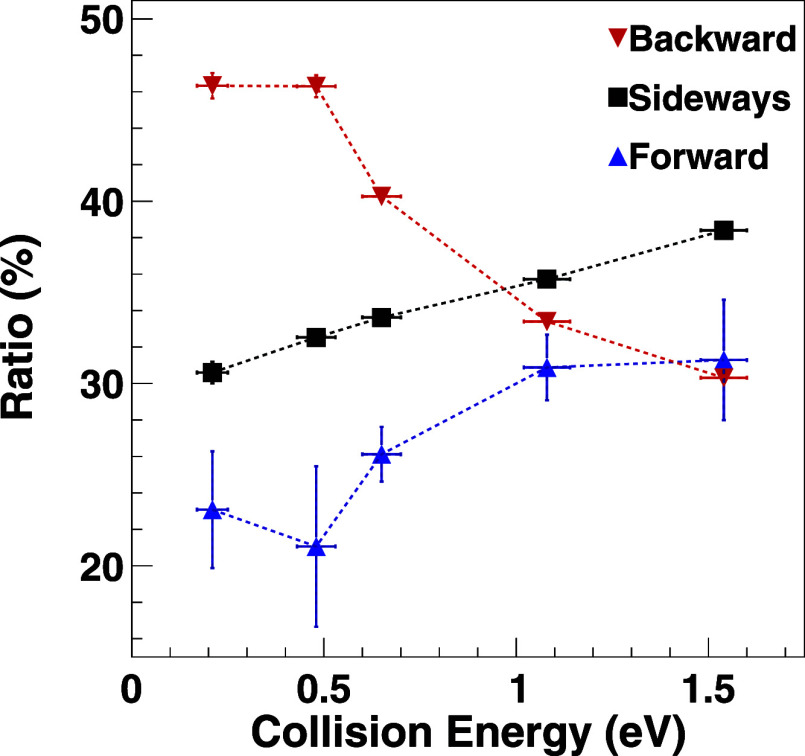
Branching of the OD^–^ product ion signal into different mechanisms, identified by the scattering angle as a function of the collision energy (see the text and Supporting Information for details). The uncertainty for sideways and backward scattered events is given by counting statistics. Forward scattered events contain the uncertainty of possible trace contaminations in the O^–^ beam.

We have quantified the contribution of forward, backward, and sideways scattering by an angle-dependent integration of the scattering images at each collision energy (see the Supporting Information for details). Specifically, we have divided the cross sections into sectors of −1 ≤ cos θ < −1/3 for backscattered products, −1/3 ≤ cos θ < 1/3 for sideways scattering and 1/3 ≤ cos θ ≤ 1 for forward scattered products. The ratio of events in each sector to the total amount of events at each collision energy gives a mechanistic branching ratio for reaction 1. Isotropic scattering would lead to equal branching ratios for all three sectors. The results are shown in Figure 2, where the uncertainty for each data point is based on counting statistics and is contained in the marker for sideways and backscattered events. Uncertainty estimates of forward scattered products are higher due to trace contaminations of OH^–^ and natural isotopes of O^–^ in the ion beam. It is evident that backward scattered products are most probable at low collision energies, while sideways scattering shows a linear increase and the highest branching ratio at high collision energies. At the same time, the fraction of backscattered products decreases with increasing energy, as is also visible in the cross-sectional data (Figure 1A–C, F–H), while forward scattering also increases.

**Figure 3:**
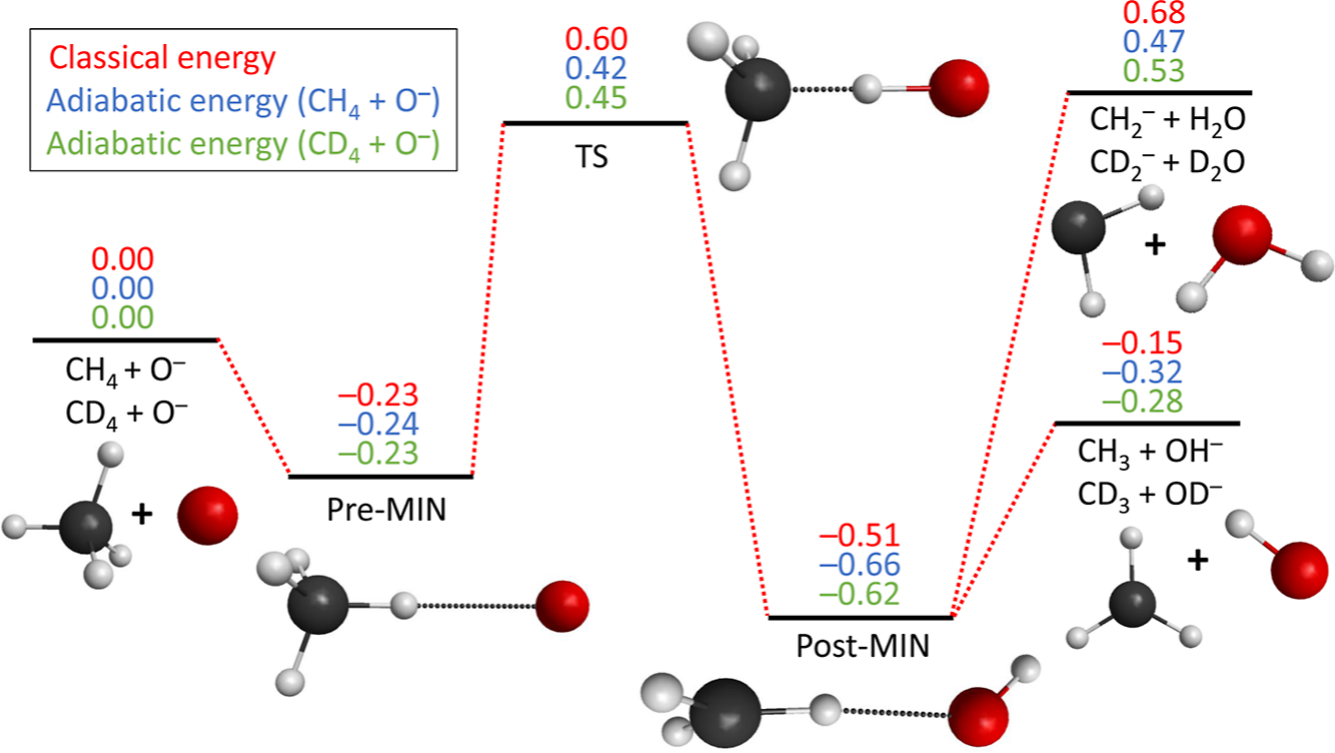
Classical and adiabatic relative energies (in eV) of the stationary points along the experimentally most relevant pathways of the O^–^ + CH_4_/CD_4_ reactions obtained at the ManyHF-based UCCSD(T)-F12b/aug-cc-pVTZ level of theory (see the Supporting Information for more details and for the challenges of determining the barrier height, which may be overestimated by the theory, as the experiment finds reactivity at lower collision energies). The reaction follows a prereaction complex (pre-MIN), a TS, and a postreaction complex (post-MIN) before proceeding to the product asymptotes.

For more information on the studied reaction, we calculated the minimum energy reaction path of both deuterium and hydrogen abstraction for the reaction of O^–^ + CD_4_/CH_4_. The results are shown in Figure 3. The reaction follows a hydrogen/deuterium-bonded prereaction complex and a TS with a low barrier, [O–X–CX_3_]^−^ (X = H/D), corresponding to the stretching of a C–H or C–D bond. Following the TS, an exit-channel minimum CX_3_···OX^–^ due to attractive ion-induced dipole interaction is found. Based on the calculations, we assume that the observed indirect scattering signatures at 0.2 and 0.5 eV might be due to the long-range ion-induced dipole attraction. We expect the entrance-channel intermediate CX_4_···O^–^ (pre-MIN) at short distances to be too short-lived for an indirect scattering signature, which is supported by a spectroscopic study.^42^ Instead, it is conceivable that the deeper exit-channel minimum may be responsible for this. With increasing collision energy, complex formation is subdued in the favor of direct dynamics with predominantly large impact parameters. This is observed despite the fact that the maximum impact parameter for ion–molecule reactions to traverse the long-range centrifugal barrier slowly decreases with increasing collision energy. Sideways and direct stripping mechanisms, in addition to the initial rebound, are observed. As most products show high angular deflection, sideways stripping with off-axis D atoms is the main reaction mechanism to produce OD^–^. The substantial geometry change from the TS to the exit-channel complex (post-MIN) is expected to induce strong forces on the reaction partners and may therefore explain the prevalence of sideways scattering over forward scattering.

The suppression of slow products at collision energies from 0.7 eV, which is visible as a distinct ring structure in the scattering images and corresponds to the missing OD^–^ product flux at high internal excitation, is likely caused by competition with a different reaction channel. A similar effect was observed previously in Cl^–^(H_2_O) + CH_3_I^43^ and O^–^ + CH_3_I.^44^ Here, an endothermic reaction channel is expected to energetically open up at around 0.7 eV relative collision energy, which is competing with the abstraction channel. Three candidates would energetically fit to a competing endothermic channel: a combined deuterium/deuteron transfer forming D_2_O + CD_2_^–^ (Δ*H* = 0.53 eV), nucleophilic substitution^24^ (S_N_2) D + CD_3_O^–^ (Δ*H* = 0.59 eV), and oxygen insertion forming CD_2_OD + D^–^ (Δ*H* = 1.01 eV). We have not observed any other ionic product other than OD^–^, and we can therefore rule out the S_N_2 and oxygen insertion reactions. The formation of H_2_O was previously observed as a combined hydrogen/proton transfer in reactive scattering experiments of O^–^ with CH_3_I.^45^ An incoming O^–^ first abstracts a hydrogen forming OH^–^. The hydroxide ion then removes an additional proton to form H_2_O + CHI^–^ in a second step. We suspect that at 0.7 eV, the exit-channel minimum CD_3_···OD^–^ in Figure 3 branches off to form D_2_O + CD_2_^–^ in a similar manner. The attractive ion-induced dipole interaction might cause an additional deuterium to swap over at high internal energy if the interaction lifetime is long enough. This causes the creation of heavy water and CD_2_^–^. Only fast scattering events, such as direct dynamics, would solely produce OD^–^. Unfortunately, possible CD_2_^–^ products are masked by the O^–^ ion beam in our experiments due to similar mass and time-of-flight. We have therefore tested in a separate experiment if reactive scattering of O^–^ with the isotopologue CH_4_ would lead to CH_2_^–^ products at a relative collision energy of 1.1 eV. Here, we were able to detect CH_2_^–^ from reactive scattering collisions on CH_4_ (see the Supporting Information). Based on this, we assign the loss of low-velocity product ions at the higher collision energies to the formation of water in a combined deuteron/deuterium transfer reaction.

The differential scattering images in Figure 1 show that the products of reaction 1 are formed with substantial internal excitation, evidenced by the separation between the kinematic cutoff and the measured distributions. The internal energy is partitioned into rovibrational excitation of either OD^–^ or the neutral coproduct CD_3_. The dashed rings shown for the three lower collision energies denote product velocities that would correspond to the excitation into vibrational levels *v* = 0, 1, and 2 of OD^–^. Additional rotational excitation of OD^–^ (rotational constant *B*_0_ = 1.24 meV^46^) would shift the distributions to lower velocities. Given the large spacing of the measured flux from the drawn vibrational levels, it is unlikely that rovibrational excitation of OD^–^ alone can explain the measurements.

**Figure 4:**
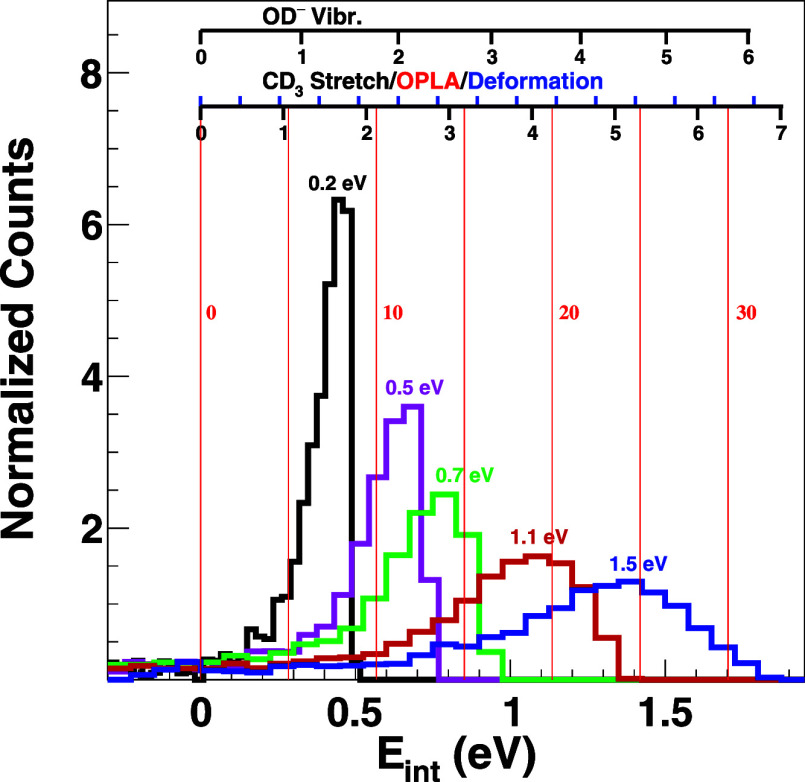
Internal energy distribution of the reaction products as a function of the relative collision energy. The vibrational levels of OD^–^ and CD_3_ products are indicated above the histograms. The three different shown vibrational modes of CD_3_ are the symmetric stretching (black tick marks pointing downward), the deformation (blue tick marks pointing upward), and the out-of-plane (OPLA) umbrella bending of CD_3_ (red continuous lines, in steps of five quanta).

We extracted the internal energy distributions from the scattering images for all collision energies. The results are presented in Figure 4. Both 0.2 and 0.5 eV show a sharp cutoff at the highest internal energy, which corresponds to near-zero velocity products. This is further indicative that low-velocity products are formed through prior complex dissociation. At higher collision energies, the sharp cutoff is relaxed toward broad internal energy distributions, as complex-mediated isotropic scattering is strongly suppressed and cannot form low-velocity products. It is interesting to note that the internal energy of products does not change depending on the scattering angle of OD^–^, but we observe constant internal energy distributions for forward, backward, and sideways scattered products (see the Supporting Information). The fractions of total available energy partitioned into internal excitation of products are presented in Table 1. The two lowest collision energies show that around 70% of the available energy is transferred into internal excitation. This fraction decreases by about 10% for higher collision energies, where direct scattering dynamics become more pronounced.

**Table 1:** Fraction of Energy Partitioned into Internal Excitation of Products as a Function of Relative Collision Energy^a^

*E*_rel_ (eV)	0.21	0.48	0.65	1.08	1.54
*f*_int_	0.72(2)	0.69(3)	0.63(3)	0.63(3)	0.62(2)

a The fractions have been calculated with *f*_int_ = 1 – ⟨*E*_trans_⟩/*E*_total_. ⟨*E*_trans_⟩ is the mean translational energy of products and *E*_total_ is the total available energy during a scattering event, *i.e.,* the sum of relative collision energy and the absolute reaction enthalpy. The fractional uncertainty is estimated by propagating the error of the mean and the uncertainty of the relative collision energies.

The vibrational levels of OD^–^ and several types of vibrational excitation of CD_3_, the symmetric stretching vibration (267.5 meV), the deformation (127.6 meV), and the out-of-plane bending (5.7 meV) (taken from refs (47–49)) are plotted above the histograms in Figure 4. The asymmetric stretching mode of CD_3_ is well approximated by the symmetric stretching vibration, as the energy difference between the two fundamental energy levels amounts to only 2.7 meV. The black tick marks pointing down on the axis indicate stretching vibrational levels, while the blue tick marks pointing upward show deformation levels. Out-of-plane bending motion is displayed in five quanta steps, drawn as red solid lines.

At the lowest collision energy of 0.2 eV, only one quantum of stretching vibration in any of the three stretching modes of OD^–^ and CD_3_ would fit well to the measured internal energy distribution. Given the finite collision energy resolution, a second quantum cannot be fully ruled out. At 0.5 eV, one quantum or at most two quanta of CD_3_ stretching vibrations would agree with the data. Substantial rotational excitation would then be required to match the measured distribution. While this scenario cannot be ruled out, the channeling of all vibrational energy into a specific stretching mode is not very likely. Instead, we consider it more probable that reactive scattering excites out-of-plane umbrella bending or the deformation mode of the neutral product CD_3_. This is based on the molecular symmetry considerations of the reactant CD_4_ and the product CD_3_.^22,23^ The abstraction of a deuterium causes relaxation of the *T*_d_ symmetry of CD_4_ to planar *D*_3h_ symmetry in CD_3_. This relaxation renders the umbrella motion and the deformation motion Franck–Condon active during reactive scattering, which induces vibrational excitation in these modes following deuterium abstraction.

It is interesting to compare the measured differential cross sections to the only previous angle-resolved scattering study of this system by Carpenter and Farrar,^23^ who used the crossed molecular-beam technique in combination with a rotatable detector at collision energies of 0.34, 0.44, and 0.64 eV. The authors identified two different mechanisms at the two lower collision energies, low impact-parameter backward scattering as the principal mechanism and forward scattering due to large impact-parameter hydrogen abstraction. The backward scattering is in agreement with the present results (Figure 1A,B). Its relative decrease with increasing collision energy was also observed previously.^23^ Interestingly, we do not observe specific forward scattered events at these collision energies but rather indirect scattering attributed to a long-lived complex. This discrepancy might be caused by the use of a rotatable detector, which implies that the slow product flux is not detectable, in contrast to the VMI technique used in the present study, which has almost uniform detection efficiency for the full solid angle. Their results at 0.64 eV also show a dissimilarity to the present work, see Figure 1C in the present work and Figure 3 in ref (23). While they only found forward scattered events, the opposite is visible in our differential scattering cross section. Given the absence of a dipole and quadrupole moment in methane, the long-range interaction is only determined by the ion-induced dipole potential, which might lead to stripping but most likely not at the large impact parameters necessary for dominant forward scattering. There is the possibility that the observed difference is hydrogen based due to a strong dynamic isotopic effect given also the strong kinetic isotopic effect^22^ observed in O^–^ + CH_4_/CD_4_.

The neutral–neutral reactions that are isoelectronic with the title reaction provide a valuable comparison for the presented ion–molecule reaction. Specifically, the reactions of atomic fluorine and the hydroxyl radical with methane were studied intensely. The state-resolved, pair-correlated differential scattering cross section of the reaction F + CD_4_ forming DF + CD_3_ was studied at a collision energy of 0.23 eV.^11^ This reaction was assumed to proceed via a direct mechanism.^50^ DF products are produced with low rotational excitation. For DF in the *v*′ = 2 vibrational level, a shift from an initial rebound mechanism toward sideways scattering was observed when higher out-of-plane umbrella excitation modes of CD_3_ were probed. For DF at *v*′ = 3, a shift from sideways to forward scattering was observed with CD_3_ products again in higher umbrella vibrational levels. This suggests again that strong direct sideways scattering as observed in Figures 1 and 2 may also correspond to higher CD_3_ umbrella excitation in the present study.

In studies of the reaction OH + CD_4_,^8,51,52^ CD_3_ products in the umbrella ground state were mainly restricted to the backward hemisphere, in line with a direct rebound mechanism. HOD products corresponding to the CD_3_ vibrational ground state were mainly found to be in the first overtone of the OD stretching mode, which implies a more of a spectator role of CD_3_ when formed in the ground state. Overall, about two-thirds of the total available energy for the reaction was transferred to internal excitation, which is very similar to the present results, as shown in Table 1.

## Conclusions

In summary, distinct reaction dynamics have been observed in crossed-beam VMI experiments of the O^–^ + CD_4_ radical ion–molecule reaction. Differential scattering cross sections for the abstraction mechanism leading to OD^–^ and CD_3_ have been measured at five different collision energies from 0.2 to 1.5 eV. Ab initio stationary-point calculations along the reaction path have been performed to support the analysis of the data. At lower energies, the abstraction mechanism follows a collinear approach of reactants, resulting in low impact-parameter rebound scattering. Additionally, the formation of a long-lived complex has been observed, giving rise to isotropic scattering with a high degree of internal excitation of the products. At higher collision energies, complex formation is suppressed in favor for a shift toward more direct dynamics, predominantly direct sideways scattering. This indicates that high-impact-parameter collisions become more important at higher energies. At higher collision energies, low product velocities are suppressed, leading to a ring-like structure in the measured differential cross sections. This suppression has been assigned to a competing endothermic reaction channel opening up at around 0.7 eV collision energy, which produces D_2_O + CD_2_^–^ in a two-step deuteron and deuterium transfer process, similar to the finding for O^–^ + CH_3_I.^45^ Throughout all investigated collision energies, the products show high degrees of internal excitation, with at least about 60% of the total available energy transferred to internal excitation. This has been attributed to the vibrational excitation of the deformation and out-of-plane umbrella vibrations of CD_3_. The obtained results have been compared to the previous experimental work on O^–^ + CH_4_^23^ and to neutral–neutral reactive scattering of F and OH with CD_4_. We hope that this work will stimulate more detailed dynamics calculations, similar to what has been achieved for closed-shell anion–molecule reactions,^53^ particularly at the low collision energies. This will allow us to achieve a more quantitative understanding of the underlying reaction pathways, the differences between the two experimental studies, and the rovibrational excitation of the two molecular products.

## Supporting Information Available

Additional computational details; angle-dependent differential cross sections of circular sectors; internal energy distributions and angular distributions for all collision energies; and time-of-flight trace showing CH_2_^–^ from reactive scattering with O^–^ + CH_4_ (PDF)
