# Supplementary material for: Imaging the Ion–Molecule Reaction Dynamics of O– + CD4
Source: J Phys Chem A. 2024 Apr 10;128(16):3078–85. doi: 10.1021/acs.jpca.3c08274 (PMC11056988; doi:10.1021/acs.jpca.3c08274)
Supplement: Supplementary file 1 — jp3c08274_si_001.pdf [file jp3c08274_si_001.pdf]

Imaging the Ion-Molecule Reaction Dynamics of $O^- + CD_4$ – Supporting Information –

Atilay Ayasli,[†] Petra Tóth,[‡] Tim Michaelsen,[†] Thomas Gstir,[†] Fabio Zappa,[†] Dóra Papp,[‡] Gábor Czakó,[‡] and Roland Wester[*,†]

†*Institut für Ionenphysik und Angewandte Physik, Universität Innsbruck, Technikerstraße 25, 6020 Innsbruck, Austria*

‡*MTA-SZTE Lendület Computational Reaction Dynamics Research Group, Interdisciplinary Excellence Centre and Department of Physical Chemistry and Materials Science, Institute of Chemistry, University of Szeged, Rerrich Béla tér 1, Szeged H-6720, Hungary*

E-mail: roland.wester@uibk.ac.at

Product channel $CH_2^- + H_2O$

In the main text, we show that product flux of the title reaction is substantially reduced at high internal energy excitation of both products. This causes a ring structure of missing flux described in the main text. We believe that this missing flux is due to a competing reaction channel, forming heavy water D_2O and CD_2^-. Possible CD_2^- products are masked in the experiment, since they have equal mass to the incident O^- beam.

To test if water may be formed at higher collision energies, we conducted reactive scattering of O^- on CH_4, as possible CH_2^- and H_2O products are not masked by O^-. We were able to detect CH_2^- product ions, as shown in Fig. S1. This strongly indicates that CD_2^- and consequently D_2O might be causing the observed missing flux. The test was conducted at 1.1 eV relative collision energy.

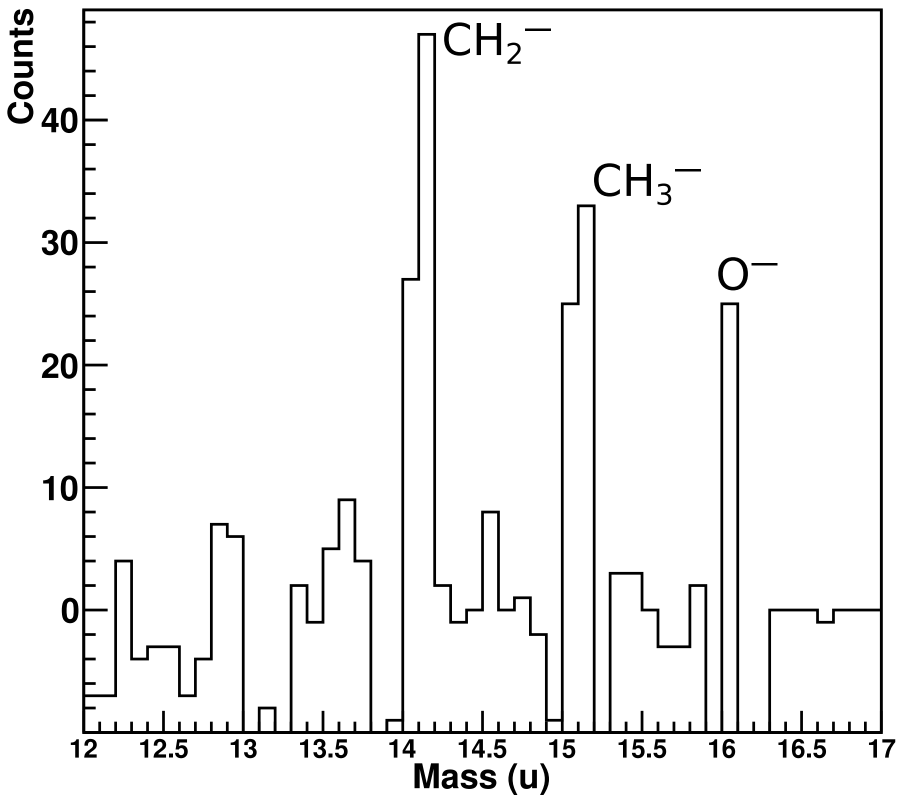

Figure S1: Detected CH_2^- products during reactive scattering of O^- on CH_4.

Additional computational details

Computing structures and energies for Pre-MIN and TS shown in Figure 3 is highly challenging as detailed below. For Pre-MIN RMP2/aug-cc-pVDZ and UMP2/aug-cc-pVDZ optimizations do not converge with MOLPRO 2015.1. Nevertheless, we manage to obtain a minimum at the UMP2/aug-cc-pVDZ level using MOLPRO 2023.2. Further UCCSD(T)-F12b optimizations fail due to ROHF convergence issues. In Figure 3 we report the relative energies for Pre-MIN at the ManyHF-UCCSD(T)-F12b/aug-cc-pVTZ level based on the UMP2/aug-cc-pVDZ structures and frequencies. Note that in the case of Pre-MIN the default-ROHF and ManyHF-based UCCSD(T)-F12b/aug-cc-pVTZ energies differ by 0.01-0.02 eV depending on the MOLPRO version (MOLPRO 2015.1 and 2023.2 use different auxiliary basis sets for F12 computations).

We obtain the TS structure at the UMP2/aug-cc-pVDZ level, however, the UCCSD(T)-F12b optimizations do not converge due to ROHF convergence issues. Therefore, we compute ManyHF-based UCCSD(T)-F12b/aug-cc-pVTZ single-point energies at the UMP2/aug-cc-pVDZ geometries and utilize UMP2/aug-cc-pVDZ zero-point energy corrections. Note that here the default ROHF-based UCCSD(T)-F12b/aug-cc-pVTZ gives 0.18 eV classical relative energy, whereas the lower-energy ManyHF reference provides higher UCCSD(T)-F12b/aug-cc-pVTZ relative energy of 0.60 eV (see Fig. 3). The ManyHF-based result is assumed to be more realistic, which is confirmed by MRCI(5,3)/aug-cc-pVTZ computations, which provide 0.41 and 0.66 eV with and without Davidson correction, respectively. Note that the large Davidson correction shows the importance of dynamic electron correlation, which is better described by UCCSD(T)-F12b than MRCI, because the latter only considers single and double excitations. The origin of this electronic structure issue of the TS may come from the fact that the first excited doubly-degenerate electronic state is only above the ground state by 0.03 eV at the MRCI/aug-cc-pVTZ level.

In the case of Post-MIN the optimizations and frequency computations converge at the UCCSD(T)-F12b/aug-cc-pVTZ level and the default-ROHF and ManyHF methods give ex-

actly the same energies. Therefore, in Figure 3 we present the UCCSD(T)-F12b/aug-cc-pVTZ relative energies based on the UCCSD(T)-F12b/aug-cc-pVTZ equilibrium structures and frequencies.

Table S1. Classical (ΔE_e) and zero-point-energy-corrected adiabatic (ΔH_0) energies (in eV) of the various product channels of the O^- + CH_4/CD_4 reactions relative to the reactants obtained at different levels of theory

	O^- + CH_4						O^- + CD_4	
	MP2/aVDZ[a]		CCSD(T)-F12b/aVDZ[b]		CCSD(T)-F12b/aVTZ[c]		CCSD(T)-F12b/aVTZ[c]	
Products	ΔE_e	ΔH_0	ΔE_e	ΔH_0	ΔE_e	ΔH_0	ΔE_e	ΔH_0
OH^- + CH_3	−0.39	−0.56	−0.15	−0.33	−0.15	−0.32	−0.15	−0.28
OH + CH_3^-	1.62	1.41	1.62	1.41	1.64	1.43	1.64	1.48
CH_3O^- + H	0.43	0.18	0.71	0.46	0.75	0.50	0.75	0.59
CH_3O + H^-	1.78	1.57	1.47	1.25	1.54	1.32	1.54	1.40
CH_2OH^- + H	1.84	1.58	2.07	1.82	2.12	1.86	2.12	1.95
CH_2OH + H^-	1.21	1.01	1.07	0.88	1.13	0.93	1.13	1.01
H_2O + CH_2^-	0.59	0.37	0.66	0.45	0.68	0.47	0.68	0.53
H_2O^- + CH_2	2.41	2.19	2.33	2.12	2.27	2.06	2.27	2.11
CH_3OH^-	−1.97	−1.81	−1.98	−1.81	−2.03	−1.87	−2.03	−1.89
CH_3OH + e^-	−2.74	−2.56	−2.67	−2.48	−2.61	−2.43	−2.61	−2.46
H_2CO^- + H_2	−0.59	−0.90	−0.48	−0.80	−0.47	−0.80[d]	−0.47	−0.69[d]
H_2CO + H_2^-	0.13	−0.11	0.16	−0.08	0.01	−0.23	0.01	−0.14

[a] Results based on RMP2/aug-cc-pVDZ geometries and frequencies.

[b] Results based on ROHF-UCCSD(T)-F12b/aug-cc-pVDZ geometries and frequencies.

[c] Results based on ROHF-UCCSD(T)-F12b/aug-cc-pVTZ geometries and frequencies.

[d] ROHF-UCCSD(T)-F12b/aug-cc-pVTZ energies based on ROHF-UCCSD(T)-F12b/aug-cc-pVDZ frequencies, because ROHF does not converge for H_2CO^- during the ROHF-UCCSD(T)-F12b/aug-cc-pVTZ frequency computation.

Energy and angular distributions

The following supporting information presents internal energy and angular distributions for the reaction $O^- + CD_4$. To obtain a mechanistic branching ratio of forward-, sideways- and back-scattered products, we have divided the differential cross-sections (DCS) into equal-sized intervals in $\cos\theta$. $-1 \leq \cos\theta < -1/3$ corresponds to backward scattered products (left hemisphere of presented DCS), $-1/3 \leq \cos\theta < 1/3$ marks sideways scattered and $1/3 \leq \cos\theta \leq 1$ represents forward scattered products. The orientation of the reactant velocity vectors is in line with the Newton diagram of Fig. (1) in the main text. The ratio between counts in each circular sector and total observed counts gives a mechanistic branching at each collision energy, presented in Fig. (2) of the main text. It is interesting to observe, that the internal energy distributions remain constant for each circular sector. This is indicative of an energetic cutoff, where CH_2^- and H_2O formation starts, instead of indirect dynamics.

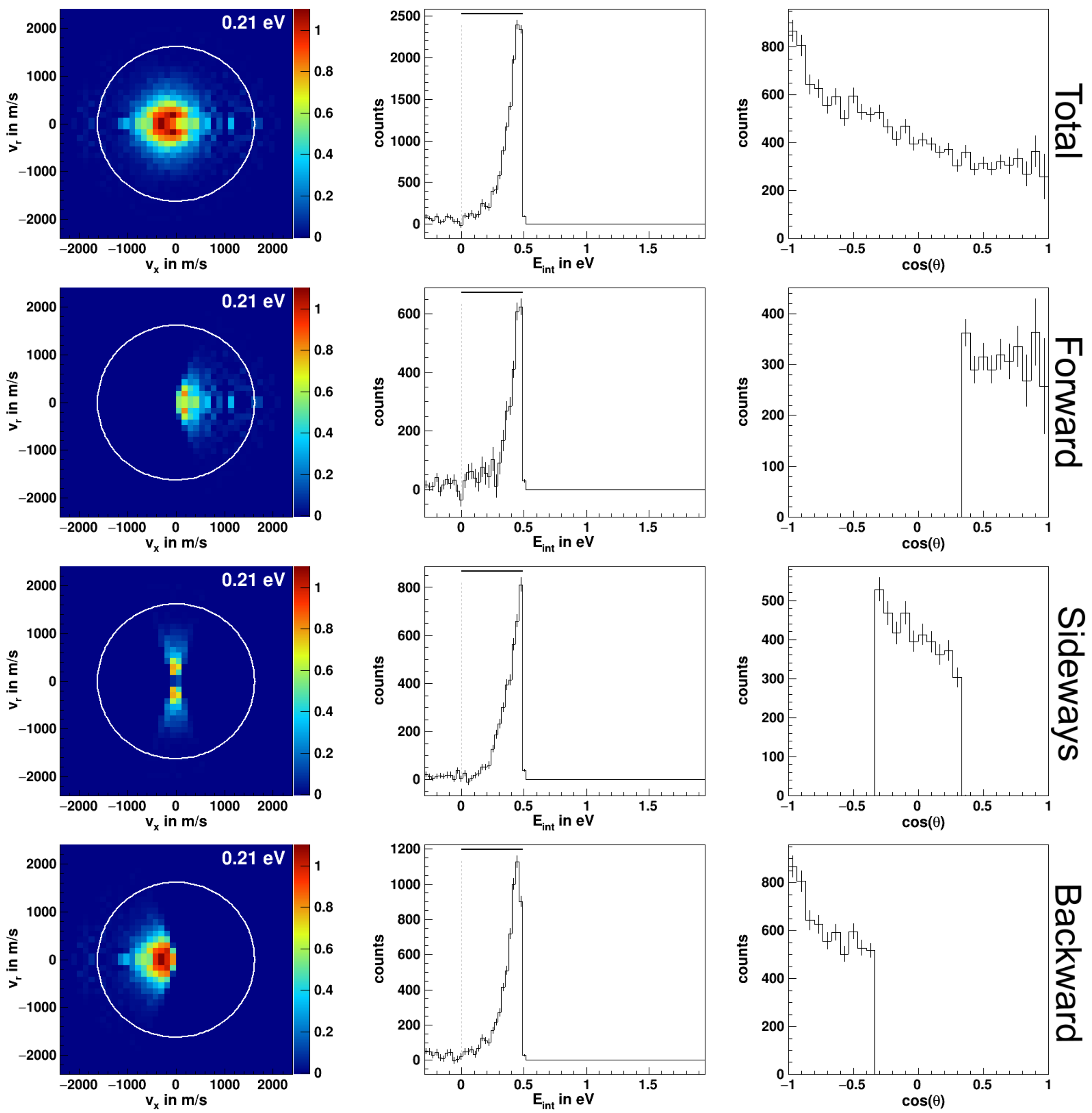

Figure S2: Experimental differential scattering cross-sections and extracted angular and internal energy distributions at a relative collision energy of 0.2 eV.

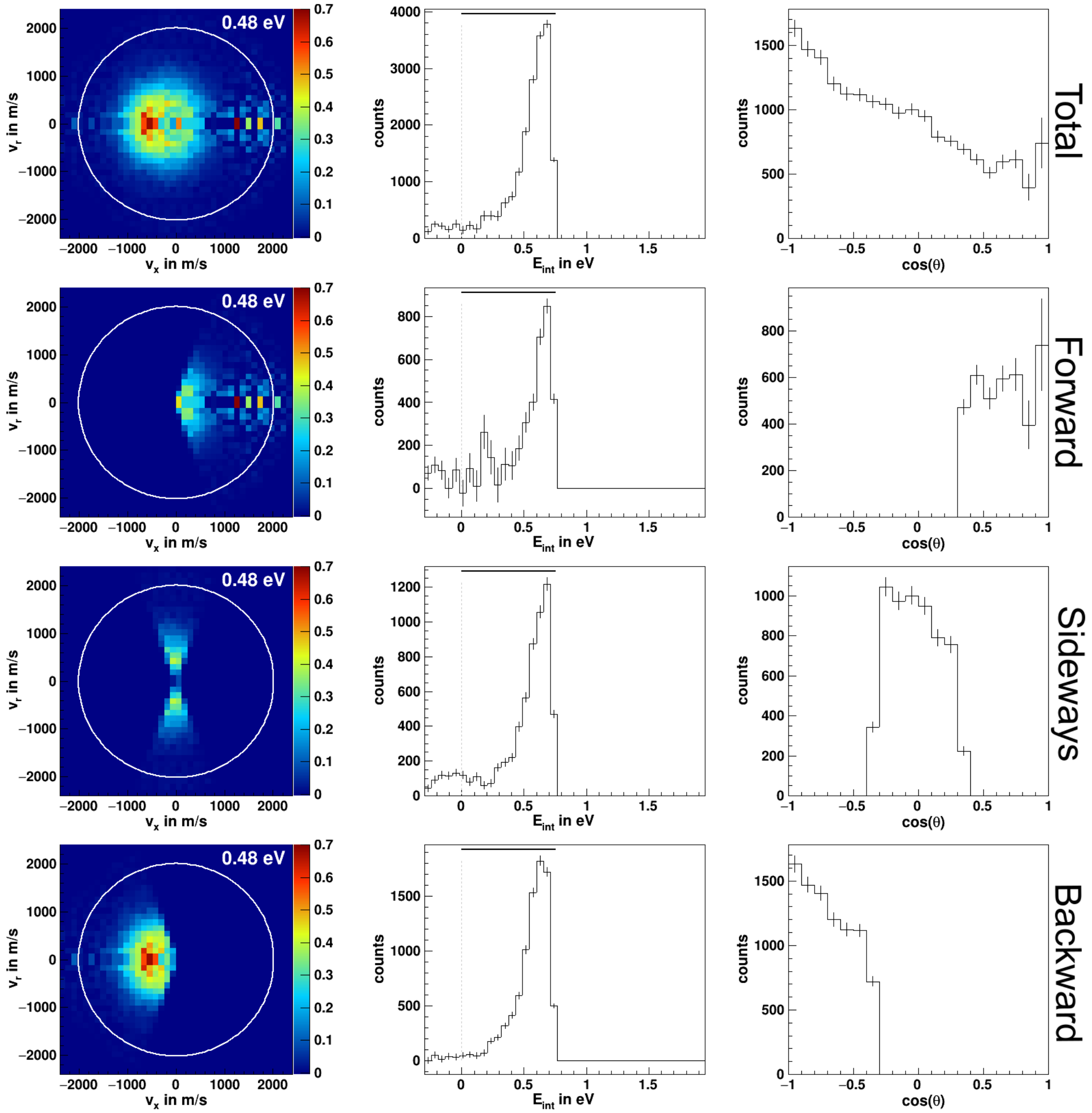

Figure S3: Experimental differential scattering cross-sections and extracted angular and internal energy distributions at a relative collision energy of 0.5 eV.

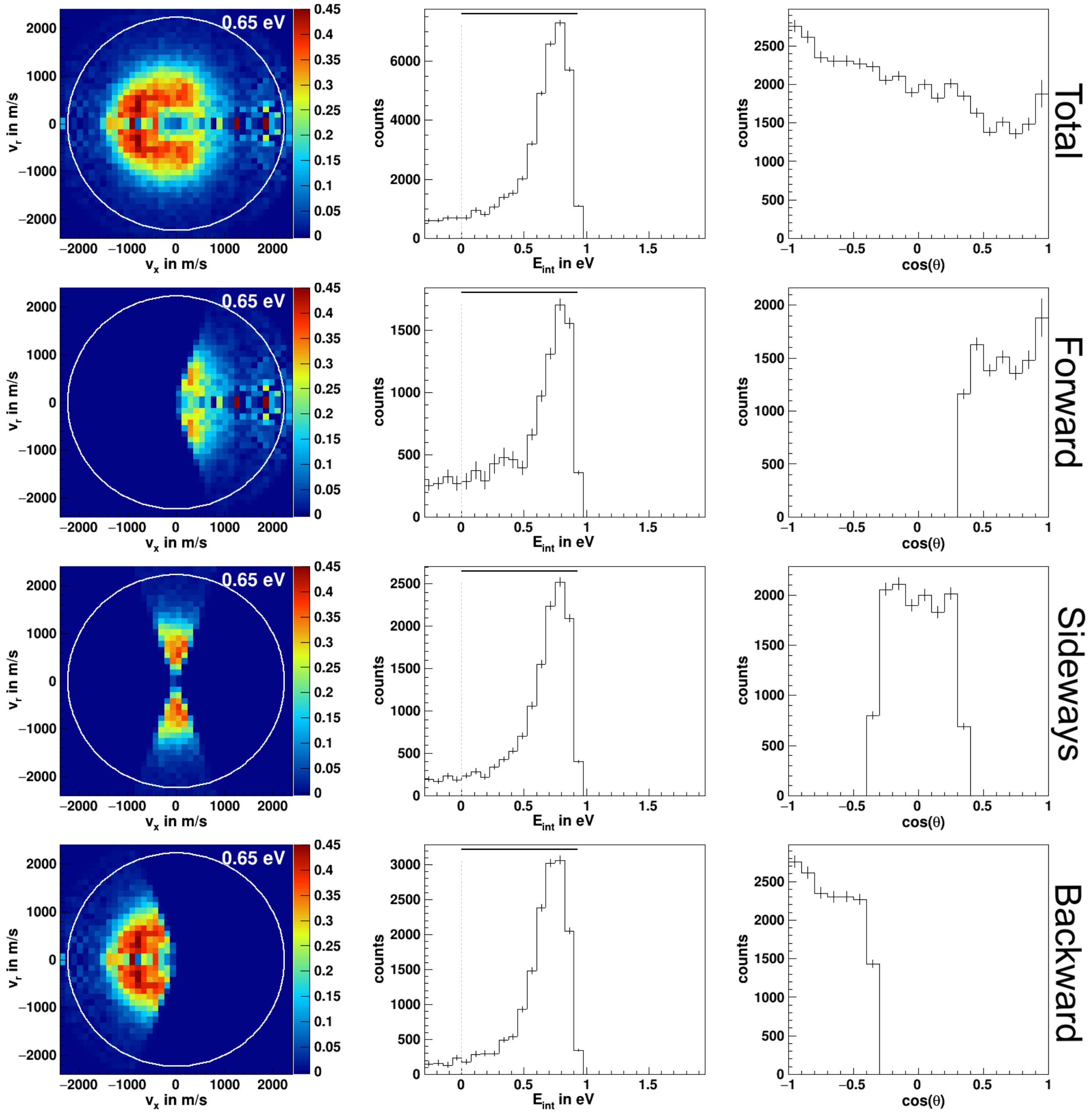

Figure S4: Experimental differential scattering cross-sections and extracted angular and internal energy distributions at a relative collision energy of 0.7 eV.

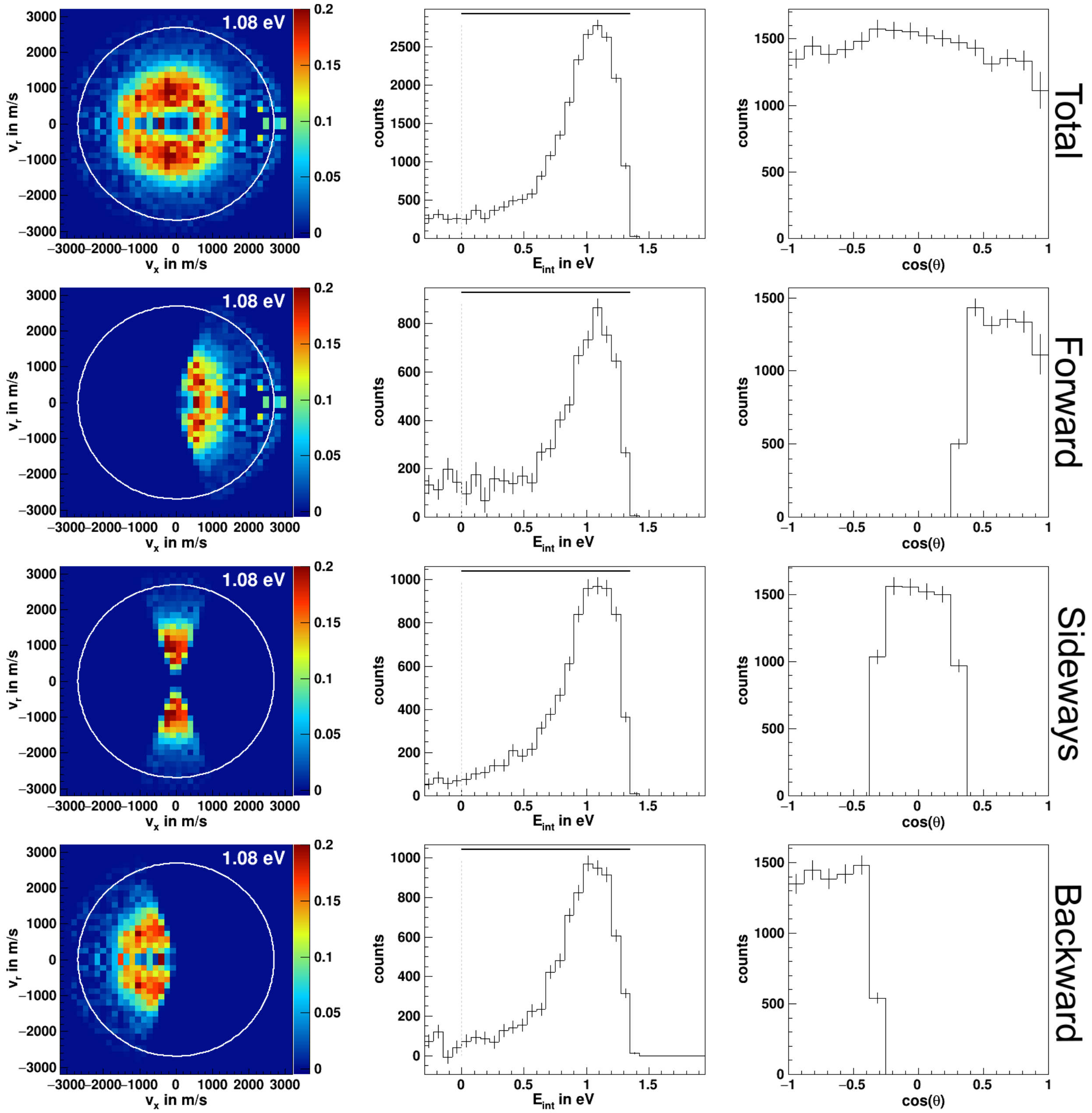

Figure S5: Experimental differential scattering cross-sections and extracted angular and internal energy distributions at a relative collision energy of 1.1 eV.

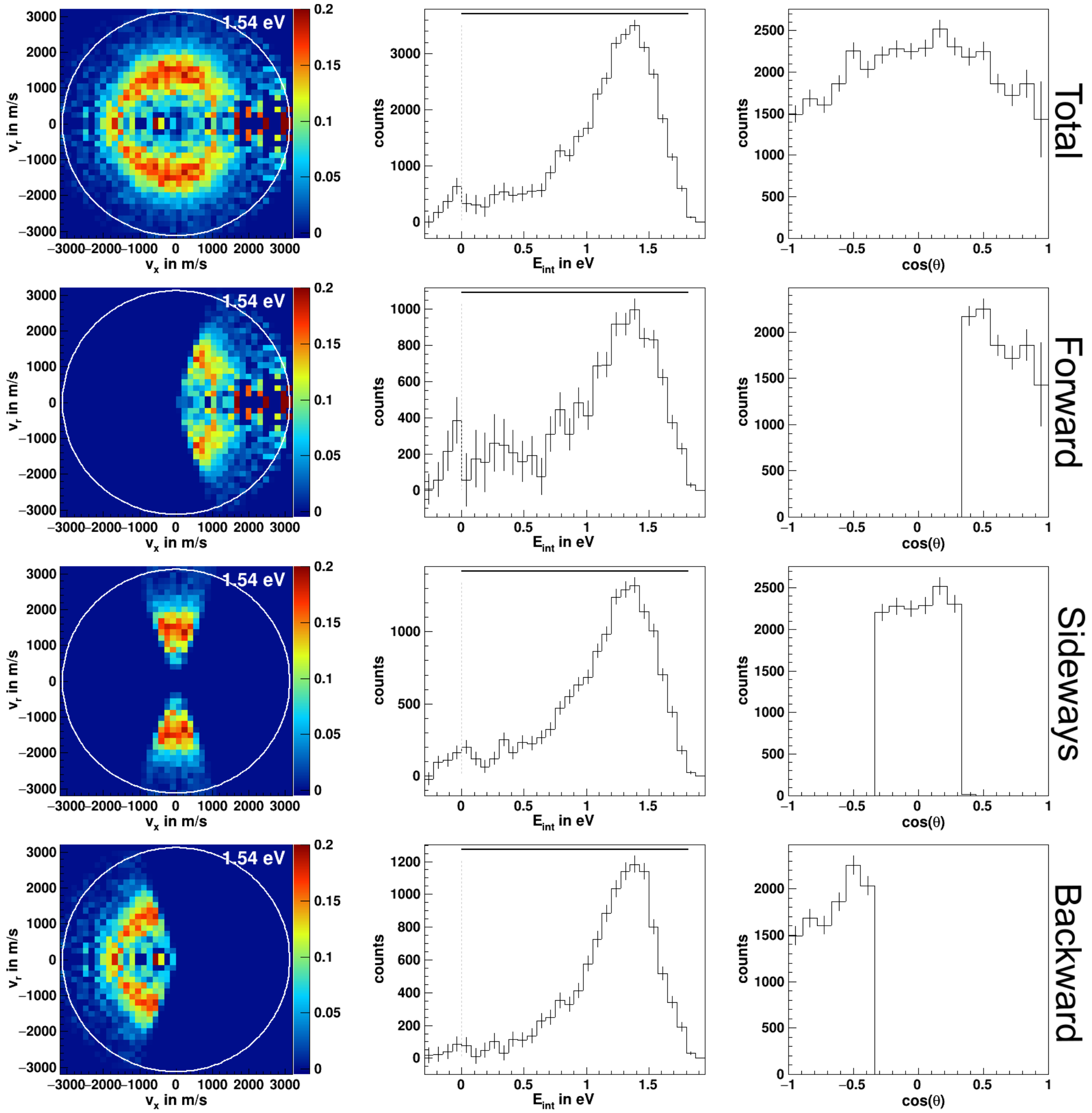

Figure S6: Experimental differential scattering cross-sections and extracted angular and internal energy distributions at a relative collision energy of 1.5 eV.
